# The Ginsenoside Exhibits Antiosteoporosis Effects in Ketogenic-Diet-Induced Osteoporosis via Rebalancing Bone Turnover

**DOI:** 10.3389/fphar.2020.593820

**Published:** 2021-01-14

**Authors:** Qi Liu, Jian Zhou, Zhou Yang, Chuhai Xie, Yan Huang, Long Ling, Yanming Cao, Hailan Hu, Yue Hua

**Affiliations:** ^1^ Department of Orthopaedic Surgery, The Second Affiliated Hospital of Guangzhou Medical University, Guangzhou, China; ^2^ Department of Spine and Joint Surgery, Nanchang Hongdu Hospital of TCM, Jiangxi, China; ^3^ Department of Orthopaedic Surgery, Southern University of Science and Technology Hospital, Shenzhen, China; ^4^ School of Traditional Chinese Medicine, Southern Medical University, Guangzhou, China

**Keywords:** ginsenoside, osteoporosis, ketogenic diet, bone loss, bone turnover

## Abstract

Ginsenoside is widely used in China for therapeutic and healthcare practice. Ginsenoside-Rb2 shows the antiosteoporosis effects in ovariectomized rodents. However, the protective effects on osteoporosis induced by ketogenic diet (KD) remain unknown. Therefore, this study aimed at evaluating the effects of ginsenoside-Rb2 on KD-induced osteoporosis. Thirty mice were randomly divided into three groups: sham, KD, and KD + Rb2. Bone microstructures, biomechanical properties, concentrations of serum bone alkaline phosphatase (BALP) and tartrate-resistant acid phosphatase (TRACP), and protein expression of osteocalcin (OCN), peroxisome proliferation-activated receptor γ (PPAR-γ), cathepsin K, and TRAP were evaluated after a 12-week intervention. The results show that KD induced significant bone loss and biomechanical impairment. Ginsenoside-Rb2 attenuated significant bone loss and maintained biomechanics in cancellous bone. The bone volume fraction increased from 2.3 to 6.0% in the KD + Rb2 group than that in the KD group. Meanwhile, ginsenoside-Rb2 effectively maintained biomechanical strengths in cancellous bone, increased serum BALP and decreased TRACP, and upregulated OCN and downregulated TRAP, PPAR-γ, and cathepsin K in the KD mice. This study demonstrated that ginsenoside-Rb2 retards bone loss and maintains biomechanics with KD. The underlying mechanism might be that ginsenoside-Rb2 inhibits bone resorption process and induces osteogenic differentiation, providing evidence for ginsenoside as being an alternative option for osteoporosis induced by KD.

## Introduction

Osteoporosis is a common disease, especially in the aged, which is manifested as deteriorated bone microarchitectures and weakening biomechanical strengths, hence fragile bone and a higher risk of fracture (Zhang et al., 2020). Osteoporosis is mainly caused by imbalance of bone metabolism, which might be affected by physical activities, diet intervention, hormonal and clinical status (Lelovas et al., 2008).

Basic researches of osteoporosis, including postmenopausal osteoporosis, glucocorticoid-induced osteoporosis, and disuse osteoporosis, rely on animal models (Komori, 2015). Recently, a new concept was introduced that osteoporosis (impaired microarchitecture of the skeletons) can be caused by certain unhealthy lifestyles, diseases, or medications. It is commonly accepted that high-fat diet can be used for obesity in rodents, which weakens the activity of osteoblasts, aggravates bone loss, and decreases bone strength in rats (Wang et al., 2017).

As an extreme high-fat and low-carbohydrate diet, ketogenic diet (KD) was initially used for treatment of epilepsy (Winesett et al., 2015). It was also applied to diverse neurological disorders (Paoli et al., 2014), endocrine disorders (Gupta et al., 2017), and cancers. However, KD has adverse effects on skeletons. Bielohuby et al. found that KD deteriorated bone growths and bone qualities in rats (Bielohuby et al., 2010). Our previous studies further confirmed that KD compromises bone mass and weakens biomechanical strengths by reducing BMSCs osteogenic capability (Wu et al., 2017; Xu et al., 2019).

Ginsenoside, which is isolated from Araliaceae family, is a major pharmacologically active compound and it contains approximately 30 active components (Cheng et al., 2012). Ginsenoside has multiple functions, such as having anticancer effects (Sun et al., 2017), prevention of neurological diseases (Zheng et al., 2018), exhibition of antioxidant (Liu et al., 2002) and anti-inflammatory (Yu et al., 2017) properties, and prevention of cardiopathy (Zheng et al., 2017). Recently, there was mounting evidence showing that the ginsenoside has antiosteoporosis effects (Yang et al., 2020). Huang et al. (2014) found that ginsenoside-Rb2 displays antiosteoporosis effects in ovariectomized rats. However, whether ginsenoside has the effects of protection from bone loss induced by KD is still unknown.

Therefore, the present study is aimed at evaluating the effects of ginsenoside-Rb2 on bone loss with KD intervention. It is assumed that (1) Rb2 attenuates osteoporosis and maintains biomechanical properties in the KD mice and (2) Rb2 effectively increases the activity of osteoblasts and inhibits the activity of osteoclasts, and rebalances bone turnover.

## Materials and Methods

### Animals, Rb2 Intervention, and Specimens Collection

Thirty female (aged 8 weeks) C57BL/6J mice were randomly divided into the sham group (*n* = 10), the KD group (*n* = 10), and the KD + Rb2 group (*n* = 10). The mice were fed with the ketogenic diet (1:3 ratio of carbohydrates to fats) (Jielikang, Shenzhen, China) in the KD and KD + Rb2 groups and with the standard diet (Laboratory Animal Center of Southern Medical University) in the sham group. Essential nutrients in the ketogenic diet and standard diet achieved a criterion of AIN-93 and were shown in our previous study (Liu et al., 2019). Ginsenoside-Rb2 (Sigma-Aldrich, Co., Ltd., United States) was supplemented by intraperitoneal injection daily at a dosage of 20 mg/kg according to the previous study which was treated effectively at the dose of 18.5 μmol/kg in the ovariectomized mice (Huang et al., 2014). All the mice were kept with a 12-h light-dark cycle and a constant temperature of 25 °C and humidity of 48%.

All the mice were anesthetized by intraperitoneal injection of 1% pentobarbitone sodium after 12 weeks of interventions. The blood was collected from the abdominal vein, and then the samples were collected after centrifuging at 1000 g. The bilateral femora were harvested for micro-CT scan, biomechanical property analysis, and histological and immunohistochemical stains, and the tibia was collected for the western blot test.

### Body Weight, Blood Glucose, Blood Ketone, Calcium, Phosphorus, and Bone Turnover Markers Measurement

The body weight in each group was collected every 2 weeks for altogether 12 weeks. The concentrations of blood glucose and blood ketone were monitored by Yicheng Blood Ketone Meter T-1 (Sentest Inc., China) and JPS-5 (Leapon Inc., China) via cutting tail veins. To limit damage, only four of all mice were selected by turns to measure the glucose and blood ketone every 4 weeks.

Using calcium and phosphorus assay kits (Beckman Coulter, Suzhou, China) to measure the calcium and phosphorus concentrations in serum, the concentrations of bone-specific alkaline phosphatase (B-ALP) and tartrate-resistant acid phosphatase (TRACP) were quantified by ELISA kits (CUSABIO, Wuhan, China).

### Bone Microstructures Analysis

The left femur was chosen for bone microstructures analysis. The distal metaphysis was scanned by a high-resolution micro-CT system (μCT 80, Scanco Medical, AG, Switzerland) with an isotropic voxel size of 10 μm, 55 kV tube voltage and 145 µA tube current. The bone trabecular analysis and 3D reconstruction were performed by the software provided from micro-CT system. The regions of interest were confined to the distal metaphysis, extending proximally 2.0 mm from the proximal tip of the primary spongiosa. The tissue mineral density (TMD), the connection density (Conn.D), bone volume/tissue volume (BV/TV), trabecular number (Tb.N), trabecular thickness (Tb.Th), and trabecular separation (Tb.Sp) were analyzed.

### Bone Biomechanical Properties Evaluation

After the micro-CT analysis, the compressive test was used to evaluate the biomechanical strengths of cancellous bone by micro-finite element analysis (SCANCO Medical AG, Version 1.13) based on the three dimensional microstructures. The simulations were done within the framework of linear elasticity which was supposed to be homogeneous and isotropic with a Poisson’s ratio of *ν* = 0.3 and Young’s modulus of material = 10,000 MPa. The simulation yielded the compressive stiffness and failure load.

**FIGURE 1:**
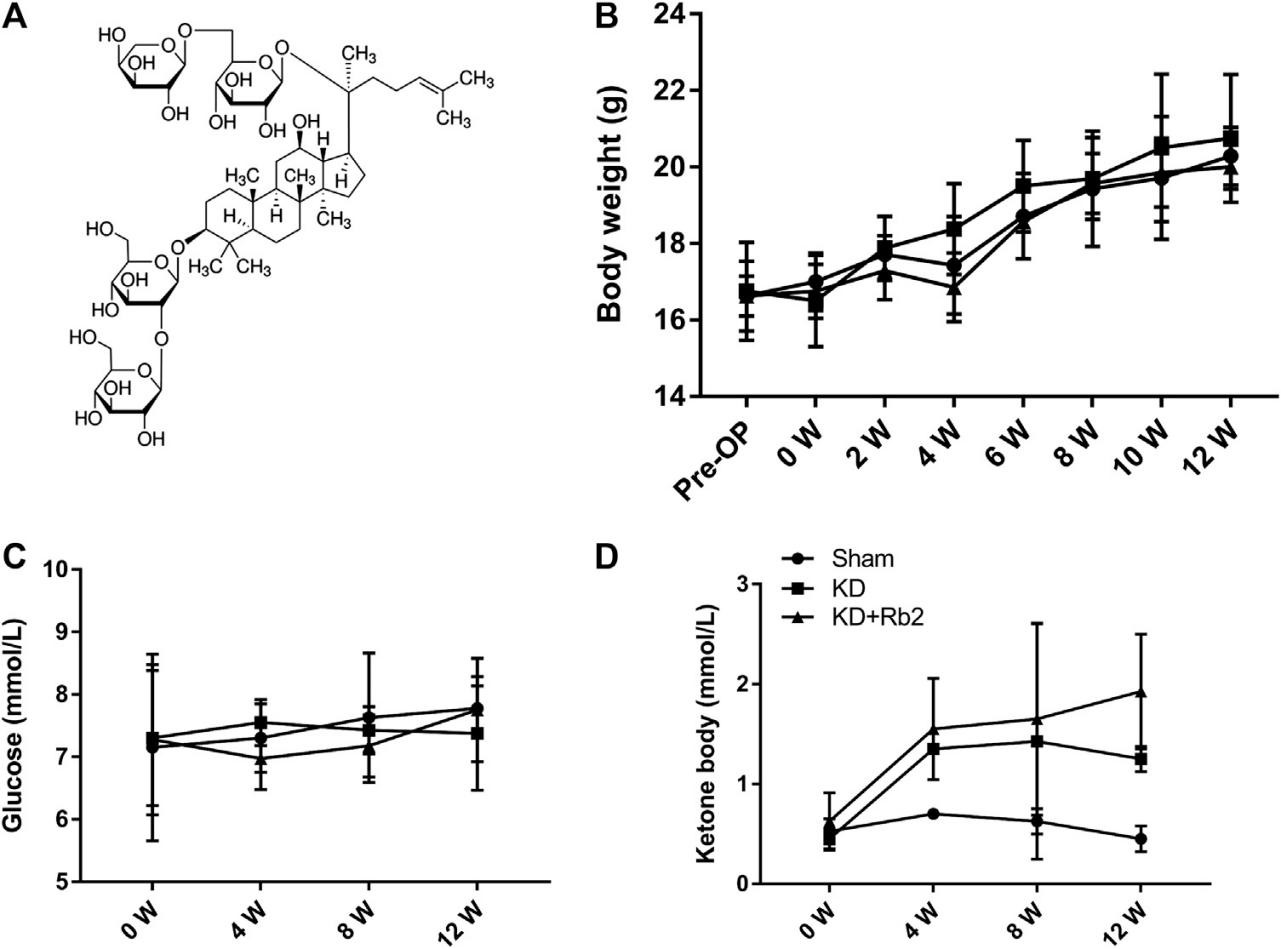
The chemical structure and general indicators. **(A)** The molecular structural formula of gingenside-Rb2. The body weight **(B)**, the blood glucose **(C)** and the blood ketone **(D)** among groups. The body weight and the blood glucose shown no difference in the sham, KD and KD+Rb2 groups, while the level of ketone body was significantly increased in the KD and KD+Rb2 groups. Data were showed as means ± standard deviation, and *P < 0.05 compared to sham group. (**B**, n=10; **C** and **D**, n=4).

The right femora were chosen to assess the biomechanical properties of the midpoint of the diaphysis by three-point bending test. Prior to examination, the bone was thawed for 1 h at room temperature. The femur was placed between two supports with a 10 mm span in a material testing machine (Instron ElectroPuls, E1000, United States), and the maximum load (Max. L), the maximum displacement (Max. D), the stiffness, and energy absorption were obtained based on the load-deformation curve with a loading speed of 2 mm/min.

### Histological and Immunohistochemical Staining

The distal femora were embedded into olefin for histological and immunohistochemical staining after microstructures analysis. The trabecula histomorphology was observed by hematoxylin-eosin stain, and the osteoblasts and osteoclasts cell activity were evaluated by osteocalcin (OCN) staining (ab41928, Abcam Biotechnology, MA, United States; the dilution ratio is 1:100) and tartrate-resistant acid phosphatase (TRAP) staining (Sigma-Aldrich St. Louis., MO, United States), respectively. In immunohistochemistry semiquantitative assays, the number of positive cells was calculated by cells per bone surface (B.S), and the staining intensity was quantified by integrated optical density per area of positive cells (IOD/area, mean density) in four different images taken at ×400 magnification, every slide with Image Pro Plus software (Media Cybernetics, MD, United States).

### Western Blot Test

Total protein isolated from the proximal half of right tibia was extracted with RIPA protein extraction reagents (Boster Biological Technology, Wuhan, China) according to the manufacturer’s protocol. The protein was separated by sodium dodecyl sulfate polyacrylamide gel electrophoresis (SDS-PAGE) on 10% gels, and the separated proteins were transferred onto a nitrocellulose membrane. Nonspecific binding sites were blocked by incubating the membrane with 5% blocking buffer (5% bovine serum albumin in Tris-buffered saline with 0.1% Tween 20 (TBS-T) for 1 h at room temperature. The primary antibodies of OCN (the dilution ratio is 1:1,000), PPAR-γ (ab41928, Abcam Biotechnology, MA, United States, the dilution ratio is 1:1,000), and cathepsin K (ab19027, Abcam Biotechnology, MA, United States, the dilution ratio is 1:1,000) were incubated with the membrane overnight at 4 °C. The HRP-conjugated secondary antibody (Boster Biological Technology, Wuhan, China) was diluted 1:1,000 and incubated with the membrane for 2 h at room temperature. Bands of immunoreactive proteins were visualized after membrane incubation with enhanced chemifluorescence reagent for 5 min. Semiquantitation of scanned images was performed using ImageJ Software. The protein expression normalization was conducted with anti-GAPDH (Abcam Biotechnology, MA, United States) to estimate the total amount of protein loaded in gel.

### Statistical Analysis

The SPSS 20.0 software was chosen to analyze the data. The body weight, the blood glucose, and the blood ketone were evaluated by repeated measurement with subsequent Student–Newman–Keuls (SNK) post hoc test. The serum markers, the parameters of bone microarchitectures and biomechanical property, and the results of immunohistochemistry are analyzed by one-way ANOVA followed by Fischer’s least significant difference (LSD) post hoc test. The statistically significant was set at *p* < 0.05.

## Results

### The Effects of Ginsenoside-Rb2 on Body Weight, Blood Ketone, and Blood Glucose

After a 12-week intervention, body weights of each group showed no significant difference at any time point (Figure 1B; *p* > 0.05). All the mice gained body weight over time, and the body weights were 20.3 ± 0.8 g, 20.8 ± 1.7 g, and 20.0 ± 0.6 g in the Sham, KD, and KD + Rb2 groups at the end of the study. Blood glucose concentration showed no difference among the three groups (Figure 1C; *p* > 0.05), while levels of serum ketone body were significantly higher in the KD and KD + Rb2 groups in comparison with the sham group (Figure 1D; *p* < 0.05). It is demonstrated that ketogenic diet effectively increases ketone body levels, and ginsenoside-Rb2 has no effect on blood ketone body as well as blood glucose.

**FIGURE 2:**
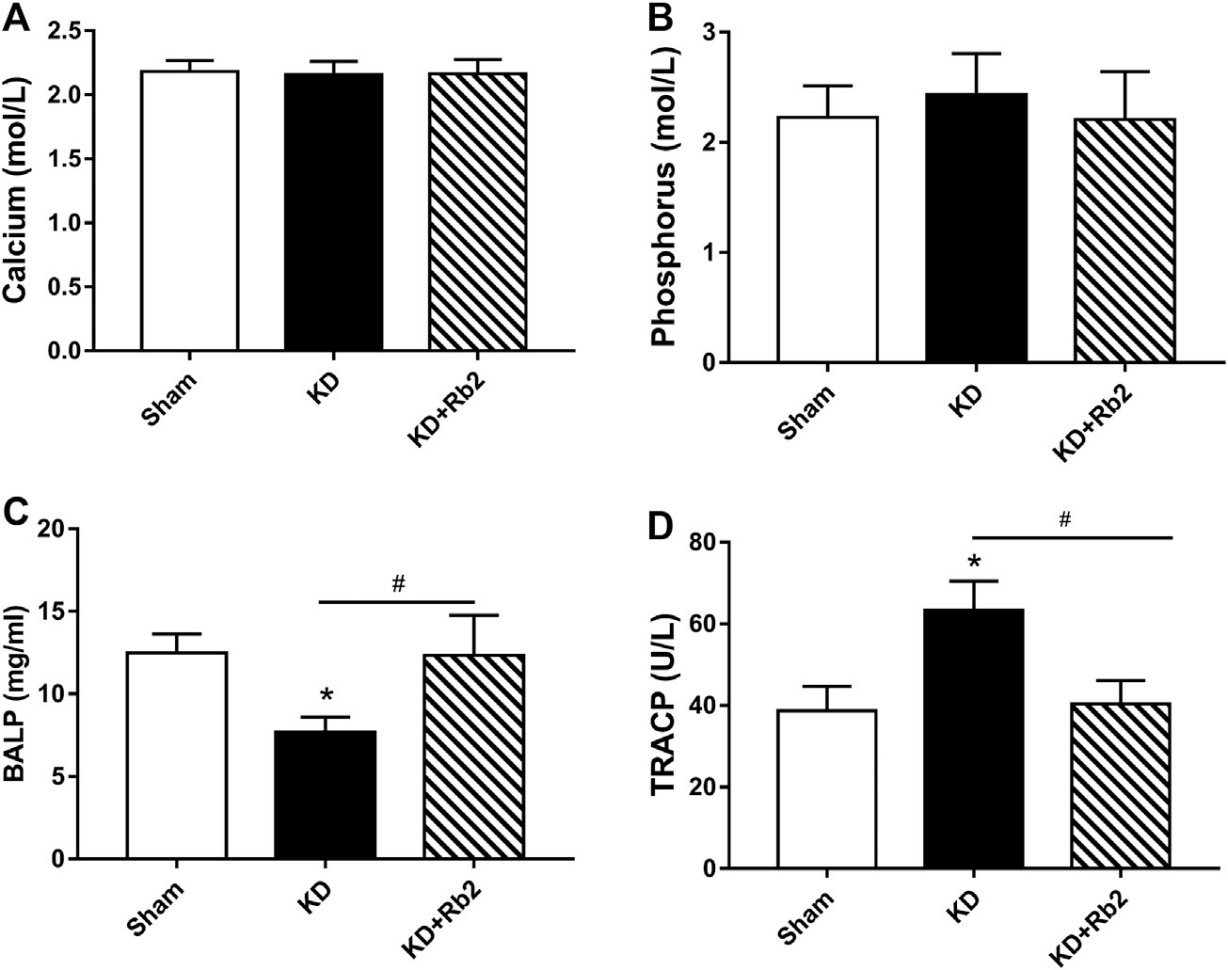
The serum markers among groups. The concentrations of calcium **(A)**, phosphorus **(B)**, BALP **(C)** and TRACP **(D)** in serum among groups. The serum calcium and phosphorus were shown no difference among groups. While, the KD significantly decreased the BALP level, and Rb2 effectively risen the concentration of BALP in the KD+Rb2 mice. Meanwhile, the TRACP was remarkably increased in the KD group, and Rb2 effectively decreased the level of TRACP in the KD+Rb2 group. Data were showed as means± standard deviation, and *P < 0.05 compared to sham group, #*P* < 0.05 between the KD and KD+Rb2 groups (n=10).

### The Ginsenoside-Rb2 Changes Bone Turnover Markers

Calcium and phosphorus in serum are the important indices for bone metabolism. In this study, calcium and phosphorus levels showed no difference among the three groups (Figure 2A; *p* > 0.05). BALP and TRACP are specific biochemical markers of osteoblast and osteoclast activities. ELISA results showed that the BALP concentration was obviously lower and the TRACP concentration was remarkably higher in the KD group than those in the sham group (KD vs. Sham: 7.70 mg/ml vs. 12.49 mg/ml and 63.37 U/L vs. 38.73 U/L in BALP and TRACP, respectively). After the intervention of ginsenoside-Rb2, the BALP concentration was significantly upregulated and the TRACP concentration was significantly downregulated in the KD + Rb2 group than those in the KD group (increased by 60.3% in BALP and decreased by 36.3% in TRACP), and it showed no difference between the sham group and the KD + Rb2 group (Figure 2B).

**FIGURE 3:**
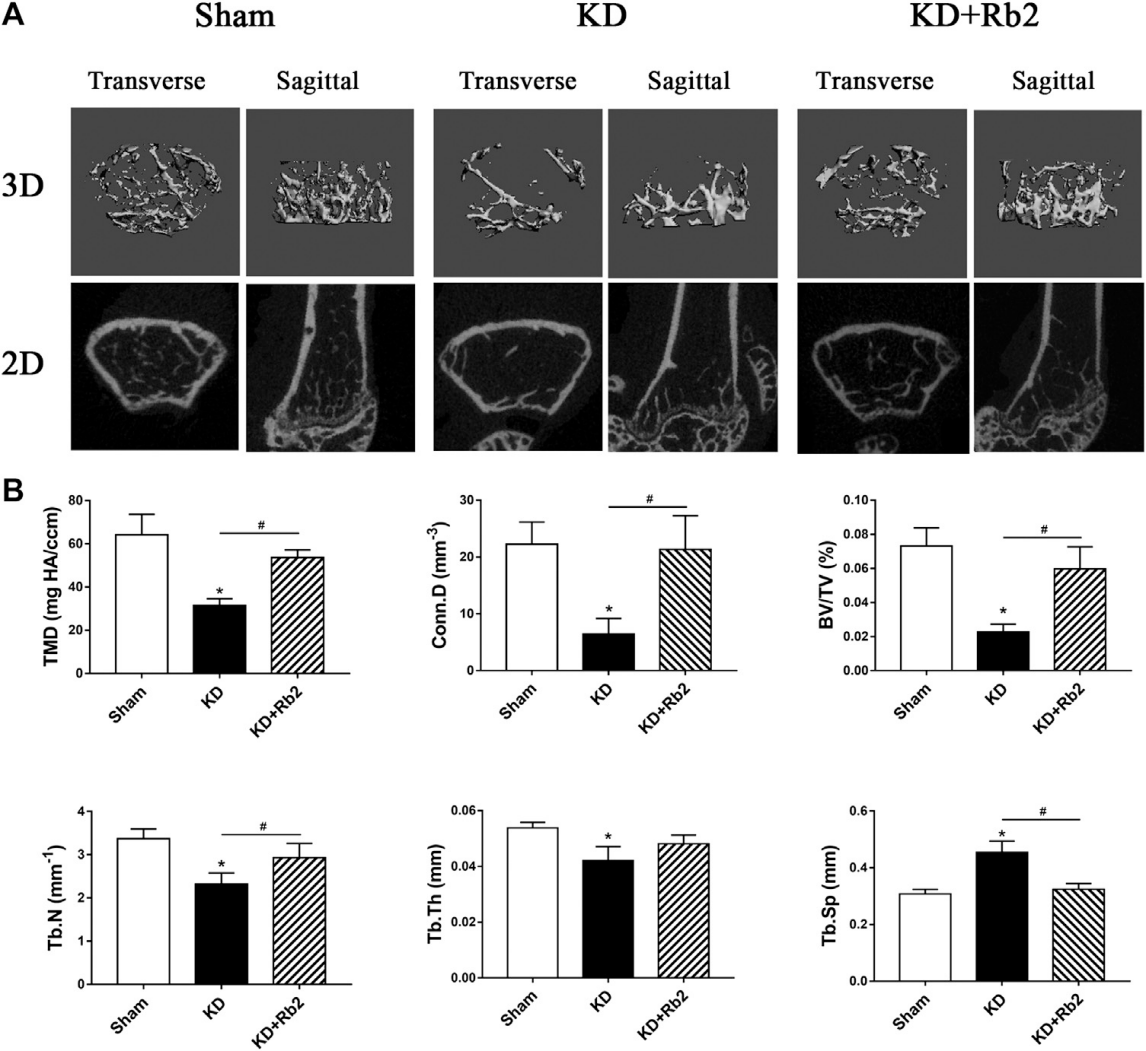
The microarchitectures of cancellous bone in the distal femur. **(A)** The three-dimensional and two-dimensional pictures of cancellous bone. **(B)** The trabecula parameters of cancellous bone. The gingenside‐Rb2 effectively ameliorated the bone loss and maintained the micro-architectures in the KD+Rb2 mice. Data were showed as means ± standard deviation, and *P < 0.05 compared to sham group and #*P* < 0.05 between the KD and KD+Rb2 groups (n=10).

### Ginsenoside-Rb2 Ameliorates the Deterioration of Bone Microarchitectures

Micro-CT data showed that microstructures of cancellous bone of distal femur were deteriorated in the KD group, with sparse and thin trabeculae (Figure 3A). TMD and Conn.D were significantly lower in the KD group than those in the sham group, with BV/TV, Tb.N and Tb.Th decreasing and Tb.Sp increasing (Figure 3B). Trabecular bone was remarkably ameliorated after the ginsenoside-Rb2 intervention. Compared with those in the KD mice, BMD and Conn.D were increased by 70.7 and 230.1% in the KD + Rb2 mice, and BV/TV, Tb.N and Tb.Th were significantly higher in the KD + Rb2 mice than those in the KD mice (KD + Rb2 vs. KD: 6.0% vs. 2.3%, 2.9 mm^−1^ vs. 2.3 mm^−1^ and 48.1 μm vs. 42.1 μm, respectively; *p* < 0.05). Meanwhile, Tb.Sp was significantly narrowed after the ginsenoside-Rb2 intervention (KD + Rb2 vs. KD: 324.2 μm vs. 453.8 μm; *p* < 0.05). Ginsenoside-Rb2 effectively alleviated destruction of bone microstructures in the KD mice (Figure 3B).

**FIGURE 4:**
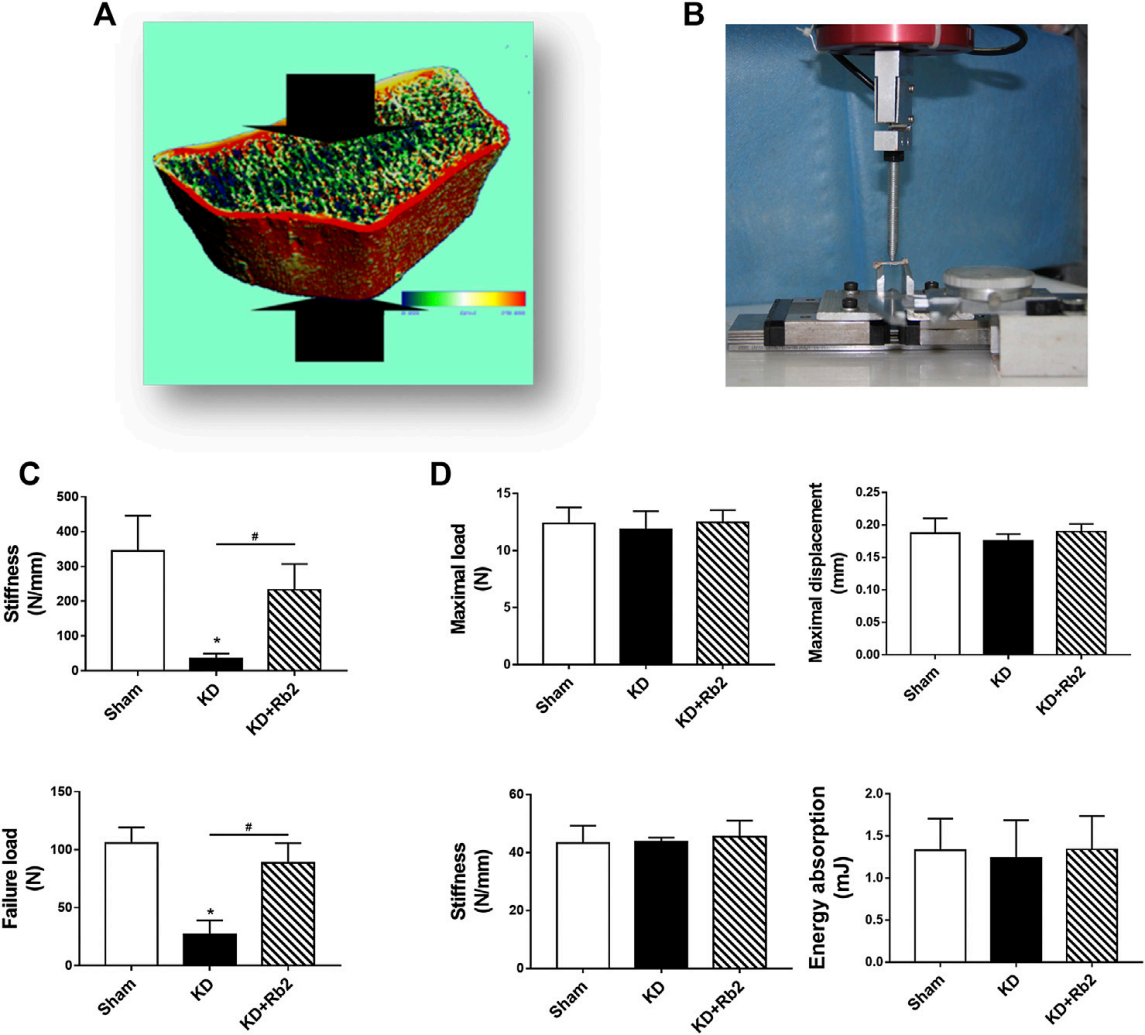
The biomechanical properties of cortical and cancellous bone. **(A,C)** The micro-FEA of cancellous bone in the distal femur. The gingenside-Rb2 effectively maintained the biomechanical strengths of the cancellous bone. **(B,D)** The three-point bending test of cortical bone in midshaft of femur. There is showed no difference among groups. Data were showed as means ± standard deviation, and *P < 0.05 compared to sham group and #*P* < 0.05 between the KD and KD+Rb2 groups (n=10).

### Ginsenoside-Rb2 Improves the Bone Biomechanical Properties in Cancellous Bone

Compression test of micro-finite element analysis (micro-FEA) in distal femur showed that stiffness and failure load of the KD group were decreased remarkably more than those of the sham group, while ginsenoside-Rb2 supplement effectively strengthened biomechanical properties in the KD + Rb2 group in comparison with the KD group (Figure 1C). The stiffness and failure load were increased from 34.7 to 232.8 N/mm and from 26.9 to 88.5 N in the KD + Rb2 group more than those in the KD group (*p* < 0.05). Meanwhile, three-point bending test of mid-shaft femur was performed to determine biomechanical status of cortical bone. The results exhibited that Max. L, Max. D, stiffness, and energy absorption of cortical bone showed no difference among the groups (Figure 4D; *p* > 0.05). These results demonstrated that the ginsenoside-Rb2 intervention effectively maintained biomechanical properties in cancellous bone.

### Ginsenoside-Rb2 Rebalances the Bone Turnover Markers in Cancellous Bone

**FIGURE 5:**
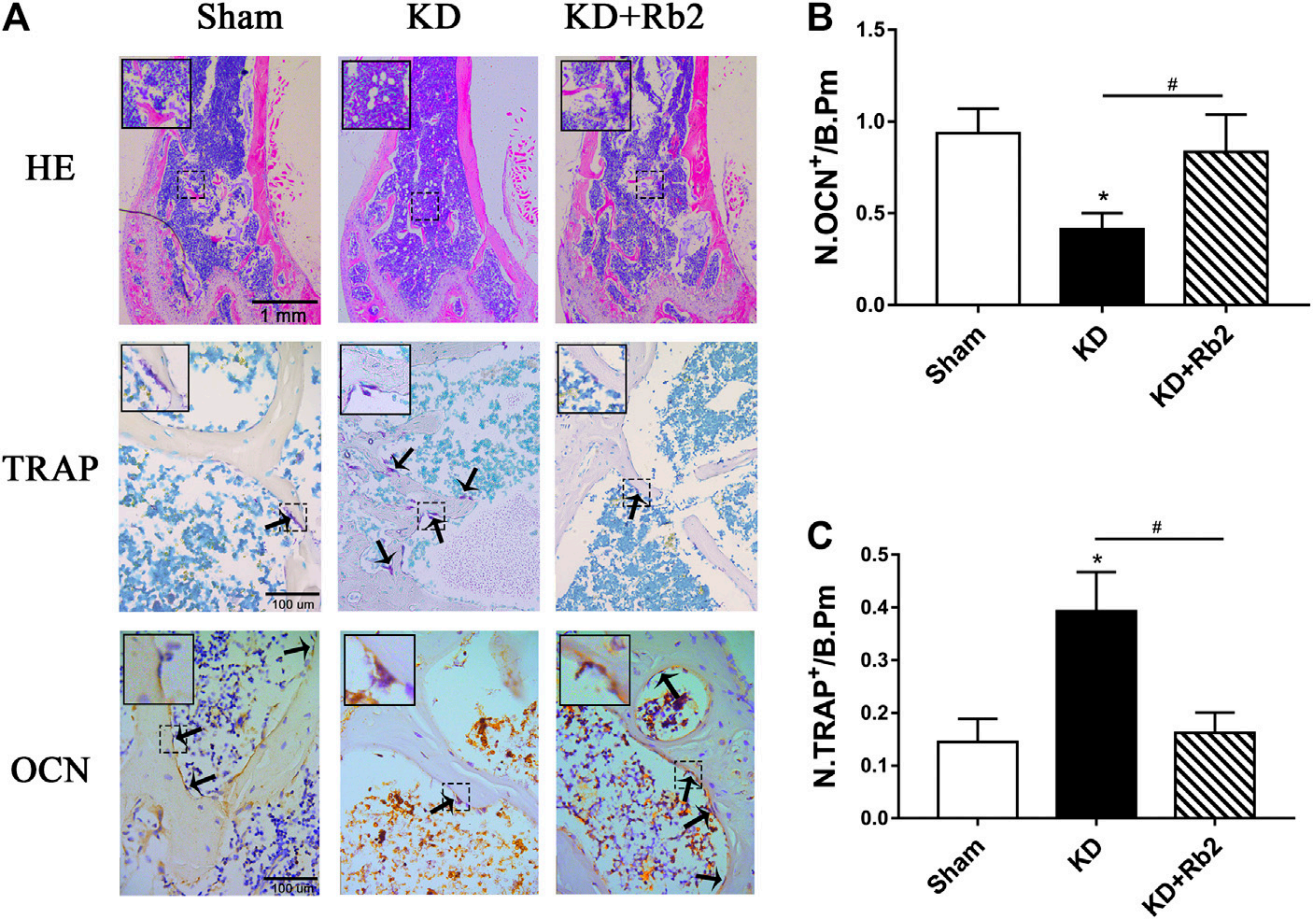
The histological and immunohistochemical staining in the distal femur. **(A)** The hematoxylin-eosin (HE) staining, the tartrate-resistant acid phosphatase (TRAP), and the osteocalcin (OCN) staining in distal femur among groups. The immunohistochemistry semiquantitative results of OCN **(B)** and TRAP **(C)**. The gingenside-Rb2 significantly increased the bone trabecula and reduced the adipose tissue in bone marrow cavity, and the gingenside-Rb2 inhibited the osteoclasts activity and increased the osteoblasts activity. Data were shown as means ± standard deviation, and *p < 0.05 was compared to sham group and ^#^ *p* < 0.05 between the KD and KD + Rb2 groups (*n* = 10).

Hematoxylin-eosin staining showed that trabecular bone was significantly destroyed in the KD group in comparison with the sham group, while ginsenoside-Rb2 effectively protected against trabecular bone reduction and remarkably reduced adipose tissue in bone marrow cavity in the KD + Rb2 group more than that in the KD group (Figure 5A).

**FIGURE 6:**
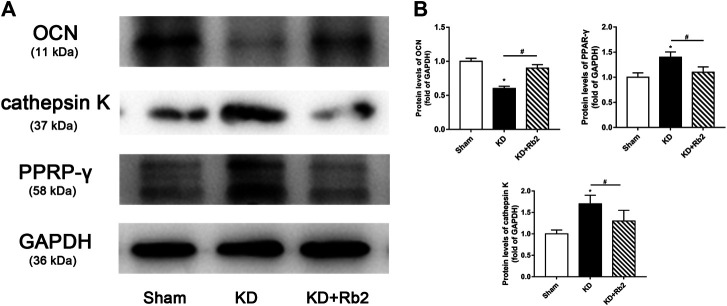
The protein expression in the distal femur. **(A)** The western blot band of OCN, cathepsin K, and PPAR-γ. **(B)** The semiquantitative results of OCN, cathepsin K, and PPAR-γ. The gingenside-Rb2 significantly increased the expression of OCN and decreased the expression of PPAR-γ in the KD + Rb2 mice. Data were shown as means ± standard deviation, and *p < 0.05 was compared to sham group and ^#^ *p* < 0.05 between the KD and KD + Rb2 groups (*n* = 10).

There was a remarkably lower expression of OCN and significantly higher expression of TRAP in the KD group in comparison with the sham group. After the ginsenoside-Rb2 intervention, TRAP-positive osteoclasts were significantly downregulated and OCN-positive osteoblasts were obviously upregulated in the KD + Rb2 group in comparison with the KD groups (Figures 5B,C). In addition, protein expression of OCN, PPAR-γ, and cathepsin K from the WB results showed that OCN expression was decreased and expression of PPAR-γ and cathepsin K was increased in the KD group more than those in the sham group, and ginsenoside-Rb2 effectively promoted OCN expression and reduced expression of PPAR-γ and cathepsin K in the KD + Rb2 group (Figures 6A,B; *p* < 0.05).

**FIGURE 7:**
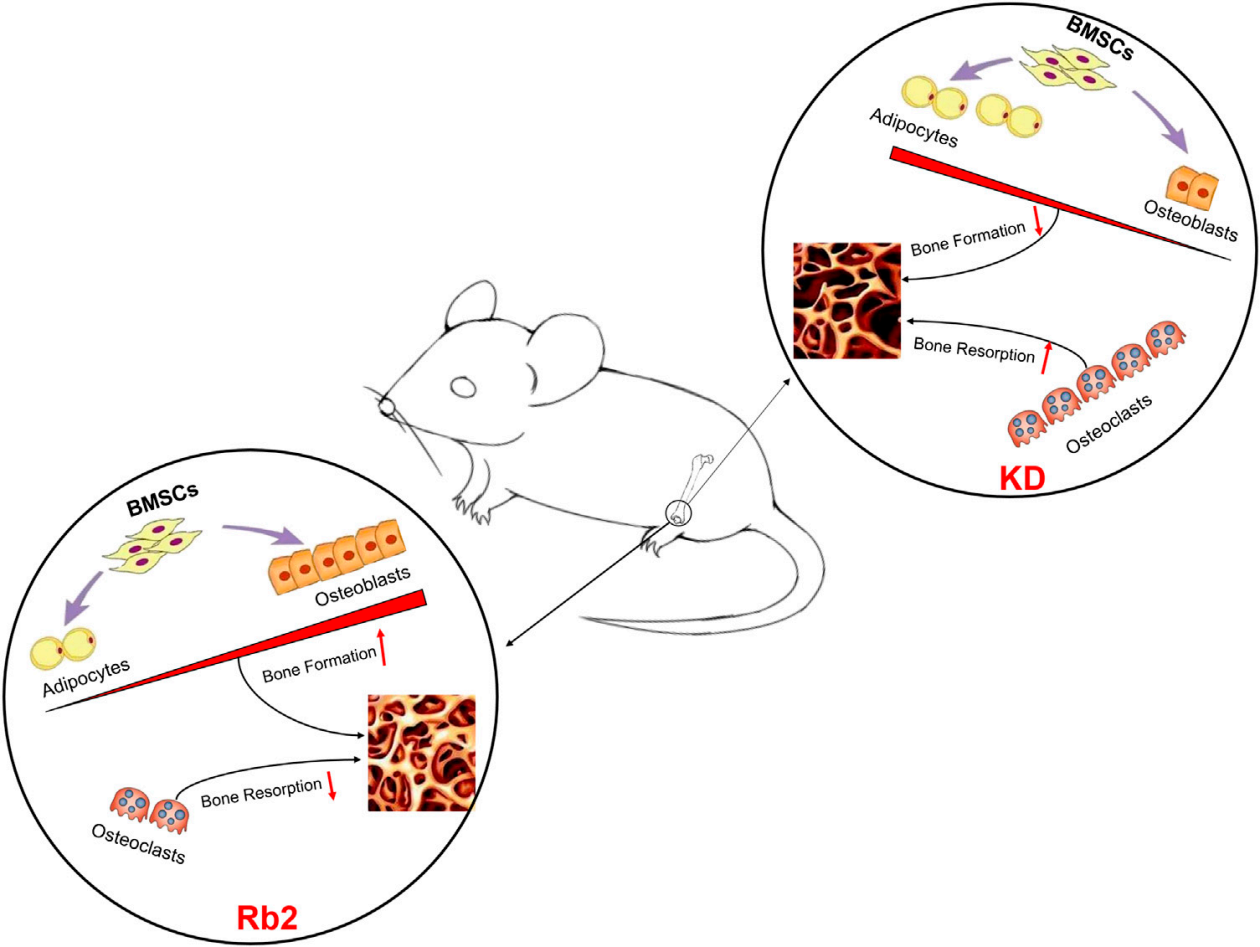
The schematic diagram of ginsenoside-Rb2 affects BMCs in mice.

These results indicated that ginsenoside-Rb2 effectively promotes osteoblast differentiation, inhibits adipogenic differentiation, and reduces osteoclast activity in the KD mice.

## Discussion

Our previous studies demonstrated that KD impairs bone microstructures and breaks the balance of osteoblasts and osteoclasts activities (Wu et al., 2017; Liu et al., 2019; Xu et al., 2019). In this study, we aim at investigating the effects of ginsenoside on bone mass, bone biomechanics, and bone turnover biomarkers with KD intervention. The results showed that ginsenoside-Rb2 effectively prevents bone loss and maintains biomechanical properties in the KD group and enhances osteoblast differentiation, decreases adipogenic differentiation, and inhibits osteoclast activity.

Ginsenoside, which is a major pharmacologically active compound extracted from *Panax ginseng*, has a lot of biological and pharmacological benefits (Gu et al., 2018). The effects of ginsenoside on bone remodeling are drawing increasing attention. Ginsenoside shows the antiosteoporosis effects by upregulating expression of intracellular osteogenic transcription factors and osteogenic related gene products, inducing osteogenic differentiation of preosteoblasts, stimulating osteoblast proliferation, and promoting bone nodule formation and matrix mineralization *in vivo* and *in vitro* (Yang et al., 2020). Ginsenoside-Rb2 is the most quantitative saponin in *Panax ginseng*, and it increases activity of ALP, promotes calcium mineralization and osteogenic mRNA expression, and exerts antiosteoporosis effects in ovariectomized mice (Huang et al., 2014). Cong et al., (2017) found that ginsenoside Rb2 inhibits osteoclast differentiation. It is confirmed that ginsenoside Rb2 significantly improves OCN expression and reduces TRAP expression in the bone tissue with KD intervention.

Similarly, the effect of bone remodeling is also reflected in bone turnover biomarkers in serum. Bone tissue is continuously removed from existing bones, which is mediated by osteoclastic bone resorption, and replaced with new bone, which is mediated by osteoblastic bone formation. Resorption rate exceeding formation rate leads to osteoporosis, which is manifested as low bone mineral density (BMD) and deteriorated bone microstructures. Bone turnover biomarkers are the landmark substances of biochemical products produced during the ongoing process of bone resorption or bone formation (Bauer, 2019). Bone formation markers, including bone isoforms of alkaline phosphatase (BALP) and osteocalcin (OCN), reflect osteoblast activity, while tartrate-resistant acid phosphatase (TRACP) is considered to reflect amount of osteoclasts (Fontalis and Eastell, 2020). In the present study, BALP level was significantly decreased and TRACP concentration was remarkably increased in the serum with KD intervention, while protein expression of OCN was decreased and expression of TRAP was increased in cancellous bone, implying that ginsenoside-Rb2 effectively maintains the balance of bone turnover.

Osteoporosis is a primary result from trabecular bone destruction, and bone microarchitectures could be referred to as a proper predictor of possible bone loss and bone quality deterioration of osteoporosis (Chappard et al., 2008). In our previous study (Liu et al., 2019), deterioration of bone microstructures by KD was observed after a 12-week intervention. Therefore, a duration of 12 weeks was determined as the time point for this study, and the microarchitectures of cancellous bone were dramatically deteriorated after the KD intervention at the time of 12 weeks. By administering ginsenoside-Rb2, destruction of trabecular bone was alleviated to some extent. Bone volume fraction was increased by 2.6 folds in the KD + Rb2 group than that in the KD group, with amount and thickness of trabecular bone increased and separation of trabecular bone narrowed. The changes of bone trabecular microstructures were consistent with the previous study of protective effects of ginsenoside-Rb2 on ovariectomized mice, which rescued the deterioration of trabecular bone microarchitectures (Huang et al., 2014).

Bone mineral density accounts for bone stiffness, and collagen is cross-linking and confers strength in tension. Therefore, biomechanical properties indirectly reflect bone integrity. However, bone strength could not be told by bone mass under some circumstances. Lin et al., (2016) revealed that aspirin showed antiosteoporosis effects with little improvement of biomechanics in high-fat-fed ovariectomized rats. In this study, we have been using micro-FE and three-point bending test to evaluate biomechanical strengths of cancellous bone and cortical bone. As our previous study presented, KD mainly affects cancellous bone, but not cortical bone (Liu et al., 2019). The results from the micro-FE showed that ginsenoside-Rb2 effectively maintains biomechanical strengths in cancellous bone. The stiffness and failure load were increased by 6.7 and 3.3 folds in the KD + Rb2 group in comparison with the KD group. Biomechanics of cortical bone exhibited no significance neither in the KD nor the KD + Rb2 group.

Bone marrow mesenchymal stem cells (BMSCs) play a vital role in osteoporosis, which exhibit a reduced capacity to differentiate into osteoblasts and an increased capacity to differentiate into adipocytes and finally cause a reduction of bone formation and an increase of marrow fat accumulation (Moerman et al., 2004; Li et al., 2015). BALP, which preferentially recognizes bone isoforms, regulates bone mineralization, and OCN, which is synthesized only by osteoblasts and odontoblast, is highly specific for bone formation (Pawel, 2018). During osteoblasts differentiation, BMSCs increase expression of osteogenic genes (such as BALP) and OCN protein (Hu et al., 2018). In our previous study, concentration of BALP in serum was measured and protein expressions of OCN in bone tissues were observed to verify the protective effects of metformin on osteoporosis animal models (Liu et al., 2019). Meanwhile, for direct BMSCs differentiation into adipocytes, peroxisome proliferation-activated receptor γ (PPAR-γ) plays a vital role in promoting adipogenic differentiation (Zhuang et al., 2016). In this study, ginsenoside-Rb2 shows its bone formation capacity by promoting osteoblasts differentiation and inhibiting adipocytes differentiation, which is presented by reducing adipocyte in bone marrow, elevating serum BALP, promoting protein expression of OCN, and inhibiting PPAR-γ expression. Meanwhile, regarding the KD mice, cathepsin K and TRACP, which are the biomarkers of osteoclast, were also decreased by ginsenoside-Rb2.

This study investigated the effects of ginsenoside-Rb2 on bone quality deterioration induced by KD. The results further confirm that Rb2 might improve bone mass and trabecular bone quality of the KD mice in part, and might rebalance bone turnover in mice with KD intervention (Figure 7). However, there are still limitations in this study. Firstly, we only used one dose (20 mg/kg) at a single time point (12 weeks). Various dose intervals with different time points of ginsenoside-Rb2 supplementation should be considered in future studies. Secondly, this study only showed protective effects of ginsenoside-Rb2 on cancellous bone, yet other vital markers for bone formation and bone resorption, such as BALP and cathepsin K, should be detected in bone tissues, and the mechanism needs further investigation. In addition, molecular and cellular level of the effects of ginsenoside-Rb2 on formation and differentiation of osteoblast, osteoclast, and adipocyte *in vitro* need to be further studied.

In conclusion, this study demonstrated that ginsenoside-Rb2 effectively ameliorates bone loss and maintains biomechanical manifestations in osteoporosis induced by KD. It enhances a transition from adipogenic differentiation to osteoblastic differentiation in BMSCs with KD intervention. Therefore, it is possible to use ginsenoside-Rb2 as a substantive alternative for treatment of bone metabolic diseases, especially osteoporosis caused by KD.

## Data Availability Statement

The raw data supporting the conclusions of this article will be made available by the authors, without undue reservation.

## Ethics Statement

The animal study was reviewed and approved by Animal Ethics Committee of Southern Medical University.

## Author Contributions

QL and YuH designed the experiments. QL, JZ, and ZY conducted the animal experiments. QL, JZ, CX, and YaH collected the samples. LL, YC, and HH completed the data analysis. QL wrote the manuscript and YuH revised the manuscript.

## Funding

This work was supported by a grant from the National Natural Science Foundation of China (No. 81574002) and the Scientific Research Projects of Administration of Traditional Chinese Medicine of Guangdong (No. 20211243 and 20201231).

## Conflict of Interest

The authors declare that the research was conducted in the absence of any commercial or financial relationships that could be construed as a potential conflict of interest.
